# Charge transfer and electronic doping in nitrogen-doped graphene

**DOI:** 10.1038/srep14564

**Published:** 2015-09-28

**Authors:** Frédéric Joucken, Yann Tison, Patrick Le Fèvre, Antonio Tejeda, Amina Taleb-Ibrahimi, Edward Conrad, Vincent Repain, Cyril Chacon, Amandine Bellec, Yann Girard, Sylvie Rousset, Jacques Ghijsen, Robert Sporken, Hakim Amara, François Ducastelle, Jérôme Lagoute

**Affiliations:** 1 CARBON NAnostructures research Group (CARBONNAGe), University of Namur, 61 Rue de Bruxelles, 5000 Namur, Belgium; 2 Laboratoire Matériaux et Phénomènes Quantiques, UMR 7162, Université Paris Diderot – Paris 7, Sorbonne Paris Cité, CNRS, UMR 7162 case courrier 7021, 75205 Paris 13, France; 3 Synchrotron SOLEIL, L’Orme des Merisiers, Saint Aubin-BP 48, 91192 Gif sur Yvette Cedex, France; 4 Laboratoire de Physique des Solides, Université Paris-Sud, CNRS, UMR 8502 F-91405 Orsay, France; 5 UR1-SOLEIL/Synchrotron SOLEIL, L’Orme des Merisiers, Saint Aubin-BP 48, 91192 Gif sur Yvette Cedex, France; 6 The Georgia Institute of Technology, Atlanta, Georgia 30332-0430, USA; 7 Laboratoire d’Etude des Microstructures, ONERA-CNRS, BP 72, 92322 Châtillon Cedex, France

## Abstract

Understanding the modification of the graphene’s electronic structure upon doping is crucial for enlarging its potential applications. We present a study of nitrogen-doped graphene samples on SiC(000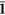) combining angle-resolved photoelectron spectroscopy, scanning tunneling microscopy and spectroscopy and X-ray photoelectron spectroscopy (XPS). The comparison between tunneling and angle-resolved photoelectron spectra reveals the spatial inhomogeneity of the Dirac energy shift and that a phonon correction has to be applied to the tunneling measurements. XPS data demonstrate the dependence of the N 1s binding energy of graphitic nitrogen on the nitrogen concentration. The measure of the Dirac energy for different nitrogen concentrations reveals that the ratio usually computed between the excess charge brought by the dopants and the dopants’ concentration depends on the latter. This is supported by a tight-binding model considering different values for the potentials on the nitrogen site and on its first neighbors.

The unique physical properties of graphene, due to the symmetry of its two dimensional honeycomb lattice and the one atomic orbital character of the low energy electronic states, make it an attractive playground for fundamental science and a promising material for widespread potential applications. However, engineering its electronic band structure in a controlled way is a crucial step towards graphene-based electronics. Inspired by semiconductor physics, the addition of electron donor or acceptor atoms to graphene has been proposed as a means to controllably shift the electronic bands of graphene. In this context, some experimental works have been performed using nitrogen^1^,^2^,^3^,^4^,^5^,^6^,^7^,^8^,^9^,^10^,^11^ or boron substitutions in the carbon lattice^12^,^13^,^14^. Nitrogen doping has been intensively studied^8^ but only few groups have reported direct visualization of band structure evolution upon nitrogen insertion with Angle-Resolved Photoelectron Spectroscopy (ARPES)^3^,^4^,^5^,^9^ or atomic-scale characterization with Scanning Tunneling Microscopy (STM)^2^,^6^,^7^,^10^,^11^ and none has combined the two techniques. A combination of a local view with macroscopic band structure determination on samples with a wide range of doping concentration is however necessary to gain a deeper understanding of the electronic effect of doping graphene with nitrogen atoms. Indeed, STM/STS is a local tool which provides information on the local densities of states close to the impurity. This is therefore a powerful technique to see resonant behavior of the nitrogen impurity. ARPES measures spectral densities of states (localized in *k*-space) extended in real space and is therefore not very sensitive to local resonances.

In a previous study^6^, we have shown that the exposure of graphene to an atomic nitrogen flux leads to the formation of nitrogen doping sites with a majority (~75%) of single subsitutional nitrogen atoms in the carbon lattice (“graphitic” nitrogen). We have evidenced that those sites display a localized donor state in the conduction band (~0.5 eV) in addition to an *n*-type doping of the graphene sheet. Contrary to what has been reported in CVD-grown samples for simple subsitutions^2^ and nitrogen pairs^7^, we did not observe any preferential sublattice for the graphitic nitrogen atoms.

In this work, we investigate the link between the atomic doping level and the electronic doping of graphene. The combination of ARPES and STM allows us to reveal the phonon contribution in STS data and the spatial variation of the Dirac point energy. The link between the nitrogen concentration and the Dirac energy is found to deviate from a rigid band model where one electron would be given by each nitrogen atom. DFT calculations as well as tight-binding calculations have already discussed this discrepancy^15^. However, it turns out that in many cases various experiments have been interpreted in terms of doping rates *n*_*e*_/*n*_*N*_ (references^2^,^3^,^5^,^16^). In this context, we explain here how a simple model taking into account the resonant behavior of the nitrogen can interpret the experimental and *ab initio* results. This deviation is explained by the formation of a localized state that captures a part of the additional electron of nitrogen.

## Results and Discussion

### ARPES and STM experiments

**Figure 1:**
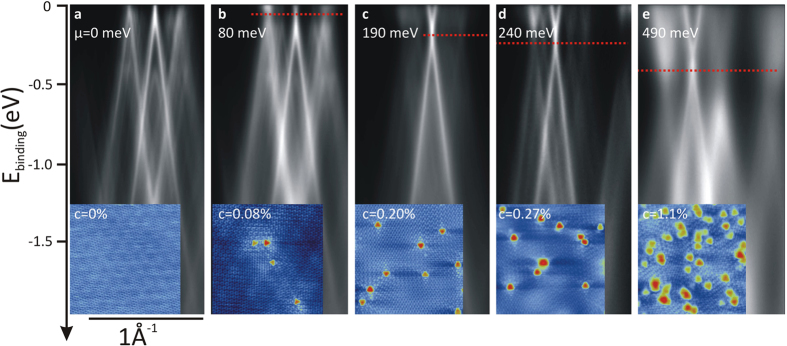
ARPES spectra of samples exposed for (from a to e) *T* = 0, 7.5, 15, 30 and 90 min. The shift of the Fermi level (*μ* = *E*_*F*_ − *E*_*D*_) is given on each spectrum. Insets are typical 10 × 10 nm^2^ STM images of the corresponding samples where the nitrogen dopants appear as red protrusions; the nitrogen concentration (*c*) is given on each image.

We have investigated the effect of nitrogen-doping on the band dispersion of graphene by ARPES experiments. In Fig. 1 we show ARPES spectra along the direction perpendicular to Γ*K* for exposure times *T* = 0, 7.5, 15, 30 and 90 min, together with typical STM images (insets), acquired on the same samples, on which individual nitrogen dopants are visible. The main cone on each spectrum comes from graphene areas whose LEED pattern is rotated by 30° with respect to the LEED pattern of the SiC substrate. The ARPES spectra display several cones for each sample. This is because the sample consists in continuous graphene sheets with rotational boundaries and the beamspot, albeit small (~50 × 50*μ*m^2^), integrates several orientations of the graphene lattice both laterally and in the stacked layers. The presence of Dirac cones in the ARPES spectra indicates that the insertion of low concentrations of nitrogen atoms in the graphene carbon lattice does not markedly modify the band structure of graphene. However, one can notice an increased width of the cones on the ARPES spectra at high concentration (Fig. 1e) suggesting that the graphene band structure starts to be affected at high doping level. This broadening of the ARPES lines correlates with the perturbation of the local density of states. Indeed, the STM image of the highly doped sample illustrates how the nitrogen atoms can scramble the local density of states (see Fig. 1e). The role of the dopants as scattering centers is clearly revealed. A careful inspection of the spectra and their Energy Distribution Curves (EDCs) (typical EDCs are displayed in Fig. S1 of the SI) shows no evidence for a gap opening in these doped graphene samples (although evaluating band gaps from multi-domain spectra can be delicate)^17^,^18^,^19^ as expected for the concentrations and the type of doping reported in this study^20^,^21^. We found that the Fermi velocity *v*_*F*_ is not affected by the doping: we measured 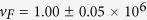 m/s on all the samples evidencing the preservation of the Dirac cone under nitrogen doping (the uncertainty on *v*_*F*_ for the highly doped sample is greater). The main effect expected from nitrogen-doping of graphene is an electronic doping *i.e.* an increase of the difference 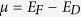 where *E*_*F*_ and *E*_*D*_ are the Fermi and the Dirac energies, respectively. From the EDCs of the spectra, we found for this energy difference 0, 80, 190, 240 and 490 meV ±10% for *T* = 0, 7.5, 15, 30 and 90 min respectively (Fig. 1). In order to link these shifts with the nitrogen concentration, we have counted the number of protrusions [*cf.* reference 6] in the STM images and we have found *c* = 0, 0.08, 0.20, 0.27 and 1.1% for *T* = 0, 7.5, 15, 30 and 90 min respectively, with a relative uncertainty of ~20% (uncertainty evaluated by taking into account the total area scanned and the measured concentration). These doping levels are proportional to the exposure time meaning that our doping technique allows good control of the doping level of the graphene samples (see Fig. S2 of the SI). In all cases, almost 90% of the nitrogen is incorporated in the graphitic form. The difference between this number and the previously published number of ~75%^6^ is due to the fact that many complex STM signatures have been identified as graphitic nitrogens close to each other, as we reported recently^22^. As also reported previously, the distribution of the dopants is almost random but the higher the nitrogen concentration, the more likely it is for the dopants to be close to each other and to interact^22^. Some effects related to these interactions are revealed and discussed below.

### XPS analysis

**Figure 2:**
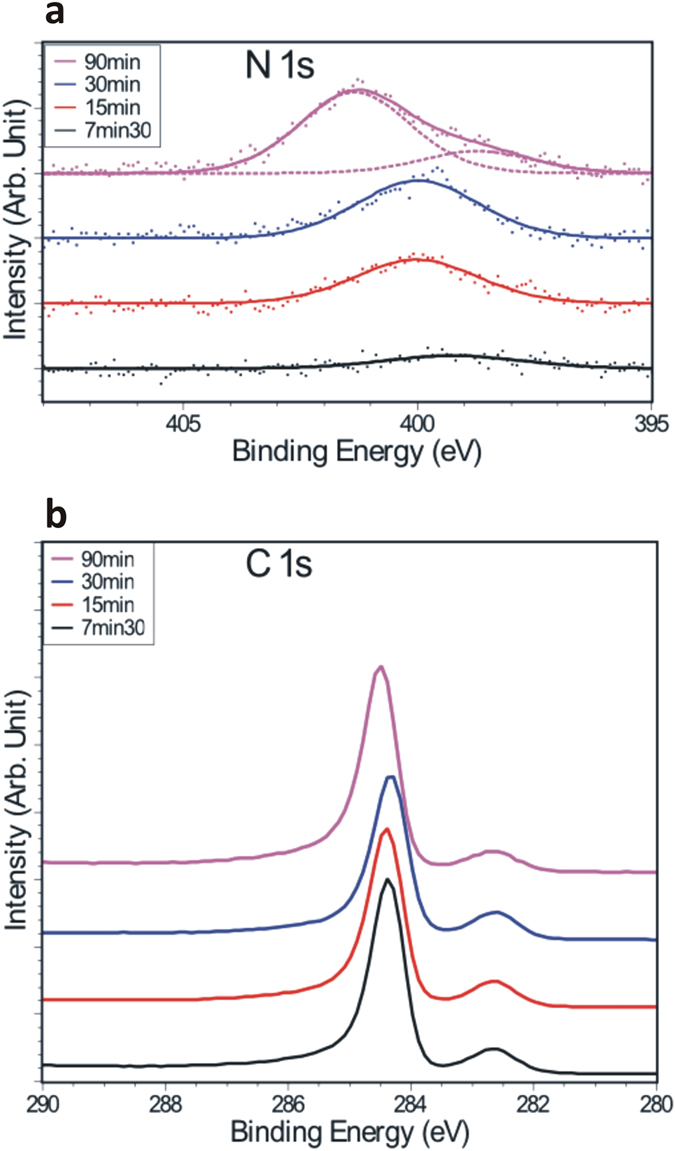
(**a**) N 1s XPS spectra for each sample (dots: experimental data, solid lines: fit, dotted lines: components). (**b**) C 1s XPS spectra for each sample.

Using XPS, we measured the N 1s and C 1s core levels spectra for each sample and show the results on Fig. 2a and b, respectively. For the lightly-doped sample (7.5 min exposure, *c* = 0.08%), although the effects on the band structure for this concentration are clear (cf. Fig. 1a as well as the ARPES/STS comparison below), the N 1s signal is at the limit of the detection threshold and a poorly-resolved peak centered around 399.5 eV can be observed. For the samples with nitrogen concentrations of 0.20 and 0.27% (15 and 30 min exposures, respectively), a peak centered at ~400.0 eV is resolved. The STM images acquired on those samples allow us to assign unambiguously these peaks to graphitic nitrogen, in agreement with the binding energies reported in the literature which vary between 400.0 eV^16^ and 402.7 eV^23^. It is to be noted that other contributions might be expected since STM revealed unidentified defects (10–15%, *cf.* reference 22). This is not seen on the N 1s spectra in Fig. 2a, probably because their concentration is below the detection limit.

For the highly-doped sample (90 min exposure, *c* = 1.1%), two contributions can be observed: the graphitic N 1s peak is found at a binding energy of ~401.2 eV (approximately 1 eV higher than that of graphitic N atoms in the samples exposed during 15 and 30 min) and another peak lies at ~398.3 eV. The ratio between the two peaks is 10–15%, similar to the ratio between graphitic nitrogen and unidentified defects observed with STM^22^. The binding energy associated to these unidentified configurations indicate that they probably involve pyridinic arrangements^3^,^4^,^24^,^25^,^26^,^27^.

We attribute the increase in binding energy of the graphitic N 1s signal for the highly-doped sample to a dependence of the spatial repartition of the nitrogen extra charge versus the nitrogen concentration (*cf.* the theoretical discussion). As already mentioned, significant variations of binding energies of the graphitic N 1s level (between 400 eV^16^ and 402.7 eV^23^) have been reported in the literature with many values comprised in this interval^3^,^4^,^5^,^9^,^24^,^25^,^26^,^28^. We believe this wide range for the N 1s binding energy is partly due to its dependence on the nitrogen concentration we evidence here.

The analysis of the C 1s spectra is delicate due to the multilayer nature of our samples as we expect our post-growth doping method (exposure to a N radical flux) to affect preferentially the top layers while XPS probes all the graphene layers. We however note the following. The C 1s spectra (Fig. 2b) of the four samples studied here display two main contributions, as already reported for the same type of samples^29^: (*i*) a small peak centered at 282.6 eV corresponding to carbon atoms belonging to the SiC substrate and (*ii*) an intense asymmetric peak at 284.4 eV associated with the sp^2^ carbon atoms of the graphene sheets. Despite the increasing shift of the Fermi level with respect to the Dirac energy (as illustrated by Fig. 1), we note that the C 1s binding energies remain almost constant at 284.4 eV for the graphitic carbon except for the highly-doped sample for which a slight shift of ~0.1 eV is observed. Again, our samples are not ideal to discuss this further but similar investigations on a monolayer sample would be of interest to clarify the link between the valence band and the core level shifts. We can also notice a very slight broadening of the C 1s peak with increasing nitrogen concentrations (from 0.5 eV to 0.6 eV for the FWHM of the graphitic peak) that could be partly due to the disorder induced by the nitrogen dopants as well as to the presence of additional C 1s components at slightly higher binding energy than the main peak and due to C-N bonds.

Finally, we note that the evaluation of the nitrogen concentration with XPS (0.1, 0.2, 0.3 and 0.5% for 7.5, 15, 30 and 90 minutes exposure, respectively) agrees only semi-quantitatively with the evaluation from STM imaging. We believe the reasons for that are (*i*) the great uncertainty in XPS due to the small amount of nitrogen and (*ii*) the fact that we expect our doping method to dope preferentially the topmost graphene layer (measured with STM) whereas XPS also probes the buried layers.

### STS experiments

**Figure 3:**
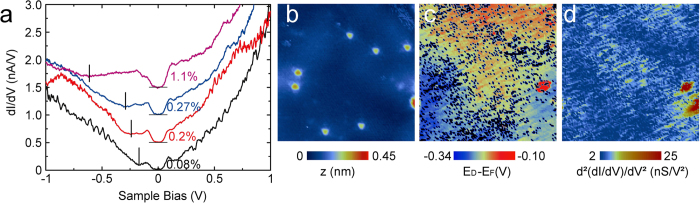
(**a**) Representative STS spectra taken on each sample (the corresponding *c* is given below each curve). The position of the Dirac point is marked by a vertical line. The curves were shifted vertically for clarity. The horizontal lines mark the position *dI*/*dV* = 0. (**b**) 12 × 12 nm^2^ topographic image obtained with *U* = 1 V and *I* = 100 pA (on the sample for which *c* = 0.20% and *μ*(= *E*_*F*_ − *E*_*D*_) = 190 meV, measured by ARPES). (**c**) *μ*(= *E*_*F*_ − *E*_*D*_) mapping (performed in the same area as in b) showing its spatial variation. Black spots mark the points where no value could be extracted (no local minimum). (**d**) Corresponding map of the curvature of the *dI*/*dV* fits at the local minimum.

**Figure 4:**
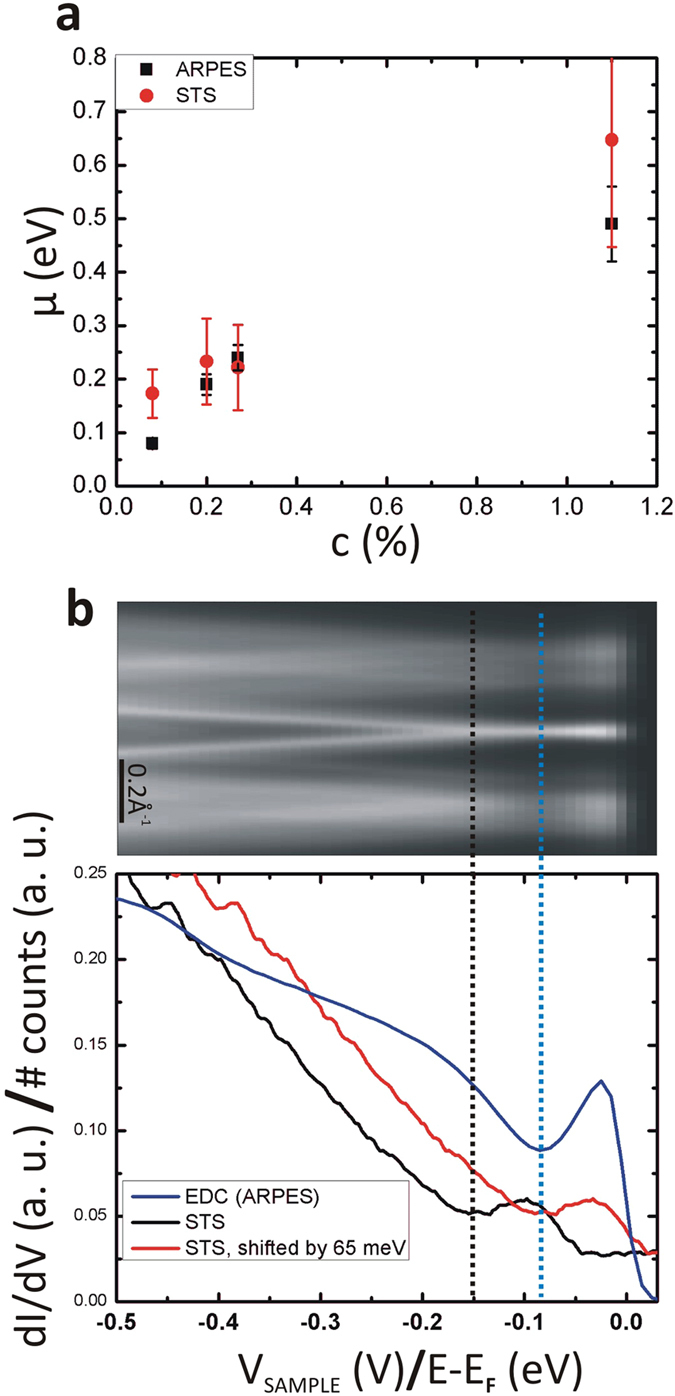
(**a**) *μ* for each sample measured with STS (red) and with ARPES (black). (**b**) ARPES spectrum taken on the lightly-doped sample (*c* = 0.08% and *μ* = 80 meV). Graph: comparison between STS and EDC data (extracted from the ARPES spectrum) revealing the importance of taking into account the apparent gap around the Fermi level in the tunneling spectra.

We now take up the analysis of the STS data. Figure 3a displays typical STS spectra taken on each doped sample, far from defects (in areas where characteristic signatures of the dopants^2^,^6^ are absent from the spectra). Around the Fermi level we observe a gap-like feature with a width of 130 ± 10 meV which is due to the inelastic excitation of an acoustic phonon of graphene^30^,^31^. We note here that the gap around the Fermi level was always clearly resolved when spectra were acquired with calibrated tips. To do so, we used the surface state of a clean Au(111) sample as the standard^32^; the spectra presented here were all acquired right after the calibration procedure. In addition to the central gap-like feature, a local minimum is observed at negative bias (occupied states), it is marked by a vertical line for each curve. When increasing the doping level, the energy position of this minimum (determined manually on individual spectra) is shifting from ~−170 meV for the sample with *c* = 0.08% to ~−600 meV for the sample with *c* = 1.1%. Therefore, we attribute this minimum to the Dirac point of the graphene samples^30^. We have noticed strong spatial inhomogeneities over the samples, as illustrated by measurements performed over the area displayed in Fig. 3b, on the sample with *c* = 0.20%. The image of Fig. 3c is extracted from a Current-Imaging-Tunneling Spectroscopy (CITS) map consiting in the acquisition of a *dI*/*dV* spectrum at each point of the topographic image of Fig. 3b: from each spectrum we have extracted the value of *μ*(= *E*_*F*_ − *E*_*D*_) (minimum of a local parabola fit) that is then used to build the image shown in Fig. 3c that displays the spatial distribution of *μ*. Interestingly, this spatial variation, that ranges on the whole image between 100 meV and 340 meV with an average of 250 meV, is comparable to the dispersion of the values of *μ* measured at different locations on the sample (both sources are reported in the error bars of Fig. 4a) which indicates that the inhomogeneity measured at the nanometer scale is the main source of variation of *μ* all over the sample. It can also be noticed that stronger inhomogeneity was observed on more doped samples as evidenced by the large variations of Dirac point energies measured on the *c* = 1.1% sample (again, reported in Fig. 4a by the error bars). This large variation of the Dirac point position is to be linked to the widening of the Dirac cones that is observed on ARPES spectra on samples with increasing doping amount (Fig. 1). It is worth noting that at certain points it is not possible to extract a value for *μ* because the fit does not display a local minimum (black dots on Fig. 3c). This happens in particular when the curvature of the spectrum is small around *E*_*D*_ and this is in turn correlated with the position of the dopants. Indeed, in Fig. 3d we report a mapping of the curvature of the spectrum around *E*_*D*_ that correlates with the position of the dopants (Fig. 3b) as well as with the black dots in Fig. 3c. We noticed in general that, similarly to what is observed on gated graphene^30^,^31^, the curvature at the local minimum is smaller when *μ* is greater, as can be seen on the typical spectra in Fig. 3a, making the determination of *μ* less accurate for heavily doped samples (another reason for the larger error bars for the more doped samples in Fig. 4a).

We now compare the ARPES and STS spectra. The averaged values of the Dirac point position found in STS together with the values found in ARPES are displayed on Fig. 4a for each sample. Despite the inhomogeneity mentioned above, *μ* is generally found to be smaller in ARPES than in STS. Although it is a debated subject^33^, this smaller value in ARPES can be explained by the apparent gap around the Fermi level seen in the tunneling spectra that is due to the absence of a phonon-mediated tunneling channel for biases 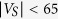 meV. Indeed, that implies that the energies of the LDOS features measured outside this central gap must be reduced by ~65 meV to obtain correct values of energy positions. This interpretation of the pseudo-gap was first put forward by Zhang *et al.*^30^ and later supported by other experimental and theoretical work^31^,^34^,^35^,^36^,^37^. This is well illustrated in Fig. 4b where the blue curve on the graph is an EDC acquired on the most lightly-doped sample (*c* = 0.08%) (computed as the angle-integration of the spectrum displayed above the graph, on the same energy scale) and the black curve is an average over tunneling spectra acquired at four different locations (at each location ten spectra were acquired) with four different tips, on the same sample, both on the same horizontal scale (the sample bias for the STS and the binding energy for the EDC). As mentioned above, the spatial inhomogeneities of the tunneling spectra are the smallest for this sample, making the comparison with ARPES data ideal.

In a first approximation, the two curves of Fig. 4b should both be (below the Fermi level *E*_*F*_) proportional to the DOS of the doped graphene (which is linear in energy close to the Dirac point). The EDC looks indeed very much like a DOS of a doped graphene sample with *μ* = 80 ± 10 meV whereas the local minimum of the STS spectrum is found at ~−150 ± 15 meV. This difference is, within the experimental error, equal to the energy of the phonon created in the inelastic tunneling process and shifting the STS curve by this energy towards the Fermi level (red curve on Fig. 4b) makes it more comparable to the EDC curve. We believe this is a strong indication that the interpretation of the gap around the Fermi level in the STS data in terms of the absence of a phonon-mediated inelastic tunneling process is correct^30^. Therefore previously reported STS spectra that did not take into account this effect should be renormalized to obtain correct values of the Dirac energy.

### Theoretical discussion

**Figure 5:**
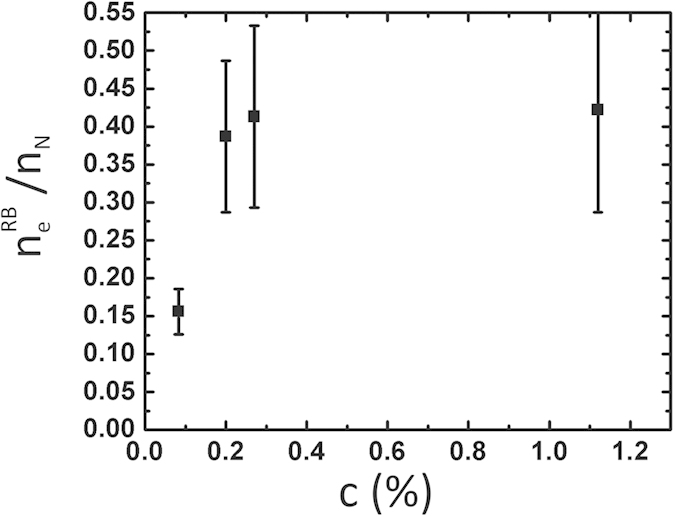
Charge transfer per nitrogen dopant (according to the rigid band model) determined from the ARPES data.

A quantity often computed is the ratio *n*_*e*_/*n*_*N*_ between the number of electrons *n*_*e*_ given by all the dopants to the conduction band of the graphene sheet and the number *n*_*N*_ of nitrogen impurities, *n*_*e*_ being determined by assuming a rigid band model where the density of states keeps the shape of pristine graphene. In the case of graphitic nitrogen, it is generally found that *n*_*e*_/*n*_*N*_ is about 0.5^2^,^3^,^6^,^5^,^16^. This is close to what we find except for the lowest concentration for which we measure 0.15 (Fig. 5). Actually the rigid band approximation is made in analogy with conventional semiconductors where donor levels below the conduction band do not perturb the conduction band and the number of electron given per each donor dopant is equal to one. This is no longer true here since the resonance induced by the graphitic nitrogen atoms is above the Dirac point. The density of states above this point is therefore larger than in pristine graphene. As a consequence the chemical potential *μ* is lower and the actual electron population of the conduction band is underestimated. In the following we call 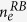 the number of electrons given to the conduction band calculated from the rigid band model. *Ab initio* calculations do confirm that deviations from the rigid band model lead to effective ratios 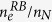 lower than one, in the range 0.5–0.7^2^. Such calculations performed with small supercells are actually not very accurate^15^, but a simple tight-binding model provides us with reliable semi-quantitative results.

A complete treatment of this problematic is behind the scope of this article and will be published elsewhere, we only outline here the method and the results of interest to us. Let *ρ*_0_(*E*) and 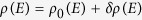 be the total densities of states without and with impurities, respectively. As a first step, we consider the case of a single impurity which is fairly easy to treat analytically within the tight-binding approximation^15^,^38^,^39^,^40^,^41^. A helpful result is that the number of *π* states below the Dirac point is not modified in the presence of an attractive potential, which is the case when replacing a carbon atom by a nitrogen one. The variation of the number of electrons is therefore strictly equal to the total electronic population in the conduction band (above *E*_*D*_ taken here as the origin of energies): 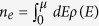. Since nitrogen brings two *π* electrons per atom instead of one for carbon, this proves that *n*_*e*_/*n*_*N*_ should be equal to unity:


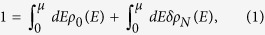


where *δρ*_*N*_(*E*) is the variation of the density of states induced by a single nitrogen atom.

To go beyond, we extend this result to the case of a finite but small concentration of nitrogen atoms. Assuming negligible interactions between the impurities, *δρ*(*E*) is now equal to *n*_*N*_*δρ*_*N*_(*E*), and the number of electrons *n*_*e*_ is equal to *n*_*N*_ = *cN*, where *N* is the number of atoms and *c* the nitrogen concentration, and we obtain:


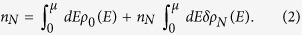


The term 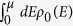 corresponds to the rigid band approximation. As usually found in the literature, this quantity (per unit surface) is given by 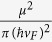, where *v*_*F*_ is the Fermi velocity. In the present tight-binding formalism, the rigid band term is equal to 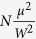, where *W* is the width of the band related to the transfer integral *t*, 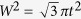. Dividing by *N* we finally find:


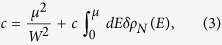


In this equation, the second term is not negligible. Close to the Dirac point, it can be approximated by *cAμ*/*W* where *A* is a constant depending on the potential of the impurity. From this equation we can determine *μ* as a function of *c*,


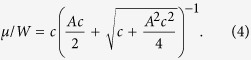


When *c* → 0, 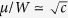, the correction to the rigid band model is negligible and the ratio 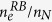 tends to unity, which is not observed. Actually this limit is obtained assuming that *A*, *i.e.* the impurity potential does not depend on the concentration. This is certainly not true: there are indications that the position of the nitrogen resonance tends to the Dirac point when *c* → 0^42^. If the variation is fast enough, the above ratio *decreases* when *c* → 0 as observed (Fig. 5).

**Figure 6:**
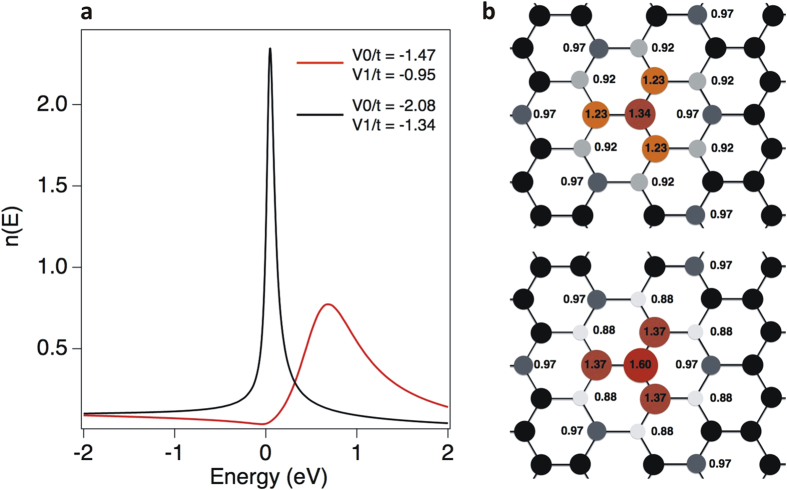
(**a**) Local density of states on the nitrogen atom for two sets of extended potentials defined by *V*_0_ on the nitrogen atom and *V*_1_ on the first neighbors. (**b**) Mulliken *π* charges around the nitrogen impurity: (top) *V*_0_/t = −1.47, *V*_1_/t = −0.95 (corresponding to the red curve in a); (bottom) *V*_0_/t = −2.08, *V*_1_/t = −1.34; t = 2.72 eV (corresponding to the black curve in a).

To illustrate this effect, we have considered a tight-binding model based on the usual description of the *π* states of graphene with a single transfer integral *t* between first neighbors equal to 2.72 eV. The local densities of states (LDOS) projected on lattice sites n(E) are calculated using the recursion method^15^. The potential due to the nitrogen impurity is assumed to reduce to on-site energies on the nitrogen site (*V*_0_) and its first neighbors (*V*_1_) which is sufficient to account for the local densities of states. They have been determined so as to fit at best *ab initio* calculations corresponding to *c* ~ 1% and are equal to *V*_0_/t = −1.47, *V*_1_/t = −0.9, respectively^15^. Assuming such parameters, the corresponding LDOS is shown in Fig. 6a with a clear resonance about 0.8 eV above the Dirac point *E* = 0. To obtain the local so-called Mulliken charges we integrate the densities of states up to the Fermi level which, at infinite dilution, remains at the Dirac point. One can object that our calculations are not self-consistent but screening effects in graphene with a Fermi level at the Dirac point are still not very well understood^41^. In this context, we find an excess p charge about 0.34 on the N atom and 0.23 on the three first neighbours (see Fig. 6b, up). Finally the integrated excess charge up to the first neighbours is equal to 1.045. Then, to simulate the motion of the resonant state, stronger potentials have to be considered. As can be seen in Fig. 6a, the local density of states on nitrogen shows a resonance very close to the Dirac point for potentials multiplied by 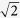, *V*_0_/t = −2.08, *V*_1_/t = −1.34. We assume that these potentials are appropriate when *c* = 0.15%. As a consequence, the excess *π* charge on the N atom and on the three first neighbours are much larger (see Fig. 6b down). In order to calculate the quantity 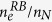, the variation of *A* with the concentration has to be determined. This can be done within the tight-binding framework using well-known exact formula^43^,^44^. For the two sets of potentials we find *A* = 5 for *c* = 1% and *A* = 70 for *c* = 0.15%. Using the previous equations, these values lead to 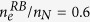 and 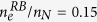 respectively, close to the experimental values. To confirm this interpretation, self-consistent calculations of the potentials as a function of the concentration are required. This will be discused elsewhere.

It is interesting to note that the calculated charge distribution reported in Fig. 6b shows a nitrogen state which is more oxidized at higher concentration (top image) in good agreement with the higher binding energy observed in XPS spectra (Fig. 2a).

## Conclusions

In conclusion, we combined STM/STS with ARPES and XPS experiments to investigate the charge distribution and electron doping in nitrogen-doped graphene. STS allows us to reveal that the Dirac energy varies spatially at the nanometer scale. The comparison of STS with ARPES shows that the STS data have to be corrected by the phonon excitation energy. The combination of the three experimental techniques together with theoretical arguments allows us to draw a picture of the doping effect of nitrogen atoms. The additional electron of nitrogen is distributed in two ways, a localized state and a delocalized state, the latter being responsible for the Dirac energy shift. This distribution depends on the concentration: at high concentration, the nitrogen atoms are more oxidized and the Dirac energy shift per nitrogen atom is larger. This understanding of electron doping in nitrogen-doped graphene paves the way to the tuning of the electronic band structure of graphene.

## Methods

The samples were grown by the confinement control sublimation (CCS) method^45^. They all consist of about 5 non-Bernal stacked layers on top of SiC. The rotational stacking decouples adjacent layers^46^,^47^,^48^. As reported earlier and as can be seen on Fig. 1a, the pristine samples are virtually undoped (electron- or hole-doped, up to a maximum of a few tens of meV)^48^,^49^. The doping technique consists in placing the pristine graphene samples in a ultrahigh vacuum (UHV) chamber and exposing them to a nitrogen radical flux produced by a remote (~30 cm) RF plasma source (MPD21 from Oxford Applied Research^50^) fed with N_2_ (purity 99.999%)^6^. Four samples were exposed for various times to the nitrogen radical flux (*T* = 7.5, 15, 30 and 90 min). The ARPES experiments were carried out at 30 K with a photon energy of 36 eV at the Cassiopée beamline of the Soleil synchrotron equipped with a Scienta R4000 electron energy analyzer; the overall energy resolution was set to 10 meV while the momentum resolution was 0.01 Å^−1^. The tunneling experiments were performed with a UHV Low-Temperature STM (4.2 K) from Omicron GmbH using electrochemically etched tungsten tips. *dI*/*dV* spectra were acquired with a lock-in detector at 710 Hz and a modulation amplitude of 24 mV. The STM images have been handled with WSxM^51^. The X-ray Photoemission Spectroscopy (XPS) measurements were performed using an Escalab 250Xi instrument from Thermo Scientific and an Al K*α* line. The emission angle was 0°. Between the different experimental setups (CCS furnace, UHV chambers for doping, ARPES and STM), the samples were transported in the atmosphere and outgassed in UHV at ~800 °C prior to manipulation (except for the XPS measurements prior to which no outgassing was possible). It has been checked by STM that this exposure to air and subsequent annealing have no effect on the samples.

## Additional Information

**How to cite this article**: Joucken, F. *et al.* Charge transfer and electronic doping in nitrogen-doped graphene. *Sci. Rep.* **5**, 14564; doi: 10.1038/srep14564 (2015).

## Supplementary Material

Supplementary Information
