# Supplementary material for: Charge transfer and electronic doping in nitrogen-doped graphene
Source: Sci Rep. 2015 Sep 28;5:14564. doi: 10.1038/srep14564 (PMC4585939; doi:10.1038/srep14564)
Supplement: Supplementary Information [file srep14564-s1.pdf]

Supporting information for: Charge transfer and electronic doping in nitrogen-doped graphene

Frédéric Joucken, Yann Tison, Patrick Le Fèvre, Antonio Tejeda, Amina Taleb-Ibrahimi, Edward Conrad, Vincent Repain, Cyril Chacon, Amandine Bellec, Yann Girard, Sylvie Rousset, Jacques Ghijsen, Robert Sporken, Hakim Amara, François Ducastelle, and Jérôme Lagoute

In Fig. 1 we display typical EDC from which the electronic doping level was measured.

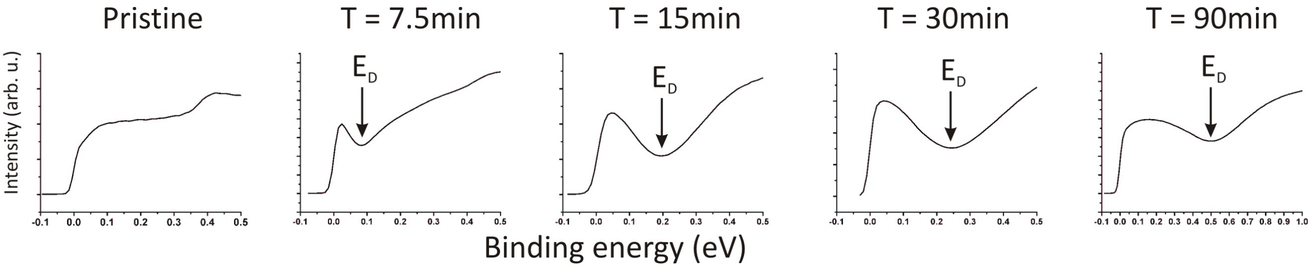

FIG. 1: Typical EDCs for the different samples from which the electronic doping level was extracted.

In Fig. 2 we report the concentration of doping sites, measured by counting the doping sites in STM images, as a function of the exposure time of the graphene samples to nitrogen flux. The quasi linear relation shows that the doping level is well controlled by controlling the exposure time.

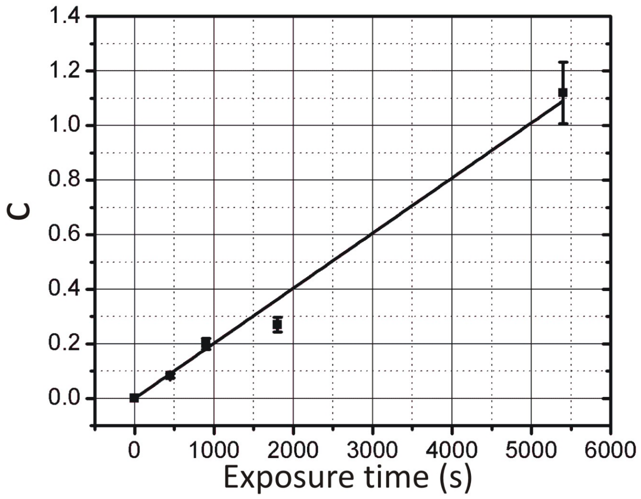

FIG. 2: Doping level (atomic nitrogen concentration; in percent) as a function of the time of exposure of the graphene samples to the radical nitrogen flux.
